# In Silico and In Vitro Assessment of Portuguese Oyster (*Crassostrea angulata*) Proteins as Precursor of Bioactive Peptides

**DOI:** 10.3390/ijms20205191

**Published:** 2019-10-20

**Authors:** Honey Lyn R. Gomez, Jose P. Peralta, Lhumen A. Tejano, Yu-Wei Chang

**Affiliations:** 1 Institute of Fish Processing Technology, College of Fisheries and Ocean Sciences, University of the Philippines Visayas, Miagao 5023, Iloilo, Philippinesf153mentor@yahoo.com (J.P.P.); lhumentejano@gmail.com (L.A.T.); 2 Department of Food Science, National Taiwan Ocean University, Keelung 202, Taiwan

**Keywords:** *Crassostrea angulata*, in silico, BIOPEP-UWM database, bioactive peptides, proteomics

## Abstract

In this study, the potential bioactivities of Portuguese oyster (*Crassostrea angulata*) proteins were predicted through in silico analyses and confirmed by in vitro tests. *C. angulata* proteins were characterized by sodium dodecyl sulphate polyacrylamide gel electrophoresis (SDS-PAGE) and identified by proteomics techniques. Hydrolysis simulation by BIOPEP-UWM database revealed that pepsin (pH > 2) can theoretically release greatest amount of bioactive peptides from *C. angulata* proteins, predominantly angiotensin I-converting enzyme (ACE) and dipeptidyl peptidase IV (DPP-IV) inhibitory peptides, followed by stem bromelain and papain. Hydrolysates produced by pepsin, bromelain and papain have shown ACE and DPP-IV inhibitory activities in vitro, with pepsin hydrolysate (PEH) having the strongest activity of 78.18% and 44.34% at 2 mg/mL, respectively. Bioactivity assays of PEH fractions showed that low molecular weight (MW) fractions possessed stronger inhibitory activity than crude hydrolysate. Overall, in vitro analysis results corresponded with in silico predictions. Current findings suggest that in silico analysis is a rapid method to predict bioactive peptides in food proteins and determine suitable enzymes for hydrolysis. Moreover, *C. angulata* proteins can be a potential source of peptides with pharmaceutical and nutraceutical application.

## 1. Introduction

Oysters are the most popular and abundantly cultured shellfish in Taiwan [1]. They are considered as one of the major species of marine bivalves, comprising 33% of the total global production [2]. In most countries, these marine bivalves are consumed as food due to its health benefits, versatility, and easy-to-prepare characteristics. Furthermore, oysters have been used as raw materials for canning, bottling, and for the production of condiments like oyster sauce and powders. Despite being highly nutritious, oysters have not gained much attention because of its inherent characteristic flavor [3]. There are also some issues associated with post-harvest processing and handling of oysters, causing its low market value. Moreover, unprocessed oyster meat has a very short shelf life and are known to pose risk to public health [4,5]. Thus, many researchers are putting much effort into the search for new post-harvest application and development of high value products from oysters.

Hypertension and diabetes mellitus type-II are two of the most common chronic diseases affecting millions of people nowadays [6]. The development of these diseases is caused mainly by certain factors. Blood pressure is regulated by the renin-angiotensin system in the body. Renin catalyzes angiotensinogen to produce a vasodilator angiotensin I. Angiotensin-I converting enzyme (ACE) is an enzyme responsible for cleavage of angiotensin I, converting it to a potent vasoconstrictor angiotensin II [7,8]. Type 2 diabetes mellitus (T2DM) is characterized by hyperglycemia due to impaired insulin secretion, as a result of degradation of incretin hormones. During meals, endocrine cells release incretin hormones such as glucagon-like peptide-1 (GLP-1) and glucose-dependent insulinotropic polypeptide (GIP) [9]. These hormones stimulate pancreatic β-cell to boost glucose-dependent insulin secretion and suppress glucagon secretion, resulting to normal blood glucose levels [10,11]. The dipeptidyl peptidase IV (DPP-IV) is a ubiquitously expressed enzyme mainly involved in the modulation of biological activity of circulating peptide hormones by breaking down the two *N*-terminal amino acids X-Pro and X-Ala. Consequently, it could result to degradation and inactivation of numerous incretin hormones with Ala as the second *N*-terminal residue, such as GLP-1 and GIP [12,13]. These mechanisms have been the basis for the formulation of therapeutic drugs targeting those enzymes. Through the years, synthetic ACE and DPP-IV inhibitors are being tried in the management of these diseases [14,15,16,17]. However, these drugs are believed to cause negative effects to human health. Daily consumption of food containing ACE and DPP-IV inhibitory peptides are known to help lower blood pressure and blood sugar to healthy levels without exhibiting undesirable effects [18]. Thus, food proteins from natural sources are now being studied as alternative therapeutic agents.

Oyster is a rich source of proteins which generally ranges from 37%–81% on a dry weight basis [19,20,21]. In general, proteins contain peptides and essential amino acids which possess specific biological activity. Biologically active peptides are short sequences of amino acids that can be released from protein precursors through gastrointestinal digestion and food processing. They provide physiological effects in the body and function as regulatory compounds with hormone-like activity [22]. Peptides need to be released from the parent protein, be ingested, be bioaccessible, and reach the target site in sufficient quantities to exhibit biological bioactivity. Recently, several studies have been focused on the generation of bioactive peptides from food proteins and their utilization as functional ingredients [23]. Previous studies revealed that oyster is a good source of biologically active peptides with antioxidant, anti-cancer, ACE inhibitory, and anti-microbial activities in vitro and show antihypertensive activity in vivo [24,25,26,27]. Having these therapeutic potential, oysters can be considered as an alternative source of peptides that can be used as an ingredient for functional foods and nutraceuticals.

One of the most common methods used for the production of bioactive peptides from food proteins is by enzymatic hydrolysis. Traditionally, the selection of enzymes suitable for liberating potent peptides are based only on literature surveys, and in vitro analyses [28]. However, this approach is costly and time-consuming. Therefore, to overcome the drawbacks of this approach, in silico technique has been proposed and utilized. This technique is useful in predicting the release of bioactive peptides from known protein sequences and selecting suitable enzyme for hydrolysis [28,29]. Furthermore, the use of this technique for screening and identification of novel bioactive peptides had shown to be much more economical and time-saving [30].

Therefore, the objectives of this study were to assess the usefulness of in silico techniques in identifying the bioactive peptides encrypted in the *C. angulata* proteins and screening for the most suitable enzyme capable of releasing these peptides. Furthermore, it aimed to evaluate the bioactivities of *C. angulata* protein hydrolysate through in vitro analysis.

## 2. Results and Discussion

### 2.1. Identified Proteins From C. angulata

**Figure 1:**
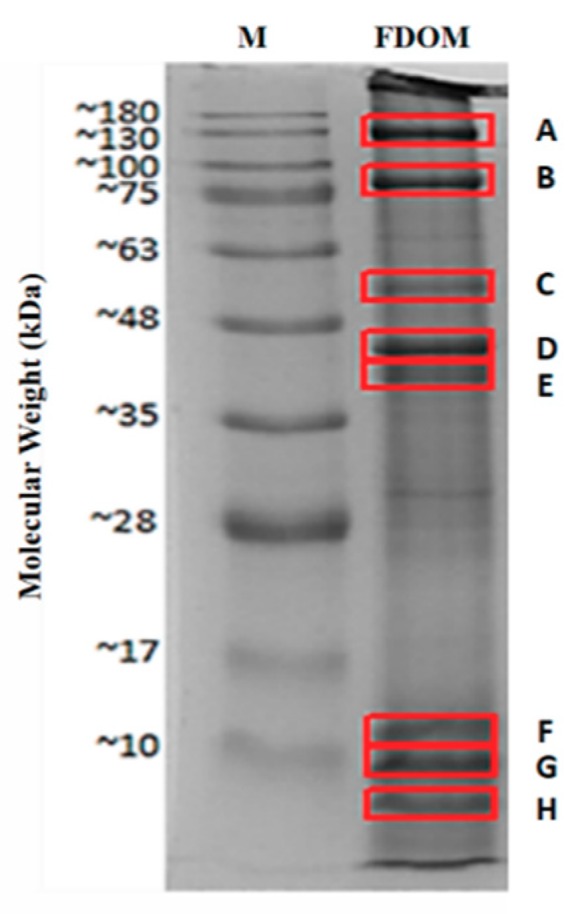
Protein patterns of Portuguese Oyster (*C. angulata*) by 12% sodium dodecyl sulphate polyacrylamide gel electrophoresis (SDS-PAGE). M: Protein marker; FDOM: freeze-dried oyster (*C. angulata*) meat.

Freeze-dried Portuguese oyster (*C. angulata*) was subjected to SDS-PAGE to separate the proteins according to their molecular weights (MWs). Among all the bands observed in the SDS-PAGE gel, the eight most distinct bands were selected for further protein identification (Figure 1). These bands were subjected to in-gel digestion and nanoLC-nanoESI-MS/MS analysis and results obtained were then matched with the information from different protein databases through Mascot database search.

**Figure 2:**
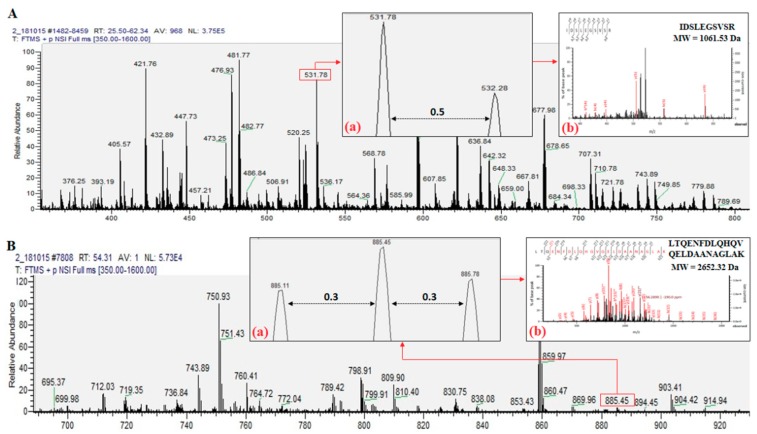
NanoLC-nanoESI-MS/MS spectra (*m*/*z* region 350 to 800 Da and 690 to 930 Da) of oyster protein band B with representative spectra of identified tryptic peptides in doubly (**A**) and triply (**B**) charged signal.

The identification of *C. angulata* proteins is based mainly on the occurrence of matched tryptic peptides from oyster (resulting from trypsin digestion) within the sequences of known proteins from the database. Based on the result from Mascot MS/MS ion search, all the identified tryptic peptides from oyster were listed as doubly or triply charged peptides. Figure 2 illustrates how the doubly charged peptide IDSLEGSVSR (MW = 1061.53 Da) and triply charged peptide LTQENFDLQHQVQELDAANAGLAK (MW = 2652.32 Da) from paramyosin (band B) were identified using the mass spectrum from nanoLC-nanoESI-MS/MS analysis. The doubly charged peptide has an observed signal *m*/*z* of 531.78 (Figure 2A). Insert (a) displays the 0.5 difference between the adjacent signals while insert (b) shows the fragmentation spectra of the identified peptide. On the other hand, triply charged peptide with an observed signal *m*/*z* 885.45 was distinguished by a 0.3 difference between the adjacent signals, as shown in insert (a) (Figure 2B). The final result and the fragmentation of this peptide was illustrated in insert (b).

Out of 16,598,945 protein sequences discovered through Mascot database search, 352 proteins under the genus *Crassostrea* were identified. In each band, one protein (highest scoring *Crassostrea* sp. protein) was selected for further screening and evaluation. From that, five proteins belonging to *Crassostrea gigas* were chosen based on their high protein scores and sequence coverage. These proteins are the myosin heavy chain from striated muscle isoform X1, paramyosin isoform X2, tropomyosin isoform X1, myosin regulatory light chain B from smooth adductor muscle isoform X2, and actin. The accession numbers, protein lengths, scores, sequence coverage, and MWs of the selected proteins were listed in Table 1.

As observed, myosin heavy chain is the largest among the five selected proteins with a theoretical MW of 220 kDa while myosin regulatory light chain B is the shortest (18.63 kDa). These proteins are both found in the muscle of oysters and other mollusks. Myosin is a contractile protein which plays a big role in muscle contraction. It is composed of six subunits: Two heavy chains and 4 light chains [31]. Paramyosin, on the other hand, is one unique protein that can be found in invertebrates such as oyster. It forms the core protein of the thick filaments of oysters which generally ranges from 3% to 9% (*w*/*w*) and constitute 38% to 48% (*w*/*w*) of the total myofibril [32]. Similarly, actin and tropomyosin are known as important proteins in oyster specifically for muscle contraction. To validate the representation of these proteins and its utilization in the subsequent enzymatic hydrolysis simulation, BLAST analysis was performed to compare the level of homology of these *C. gigas* proteins towards its *C. angulata* counterparts. BLAST analysis of myosin essential light chain protein from both oyster species resulted in 157/157 (100%) identities, 157/157 (100%) positives and 0/157 (0%) gaps, indicating total similarity of these two *Crassostrea* species in terms of their protein sequences. Thus, the identified *C. gigas* proteins were used to represent the proteins of *C. angulata* in the subsequent in silico analysis.

### 2.2. In Silico Prediction of Potential Bioactivities

In silico analysis of oyster proteins by BIOPEP-UWM database revealed that all five selected proteins are good precursors of biologically active peptides, predominantly with DPP-IV and ACE inhibitory activities, with a total of 2179 and 1391 peptides, respectively (Table 2). Most of the reported DPP-IV inhibitory peptides contain P (proline) and/or hydrophobic amino acids in their sequences. GP and PG sequences are observed to be most frequently found in meat and fish [33]. On the other hand, proteins with hydrophobic amino acid residues (W, F, Y, or P) and positively-charged group (R or K) at the *C*-terminal positions or branched aliphatic side chains (V and I) at the *N*-terminal positions are known to possess strong ACE inhibitory activity and most of these peptides contain 2–12 amino acid residues [28].

Most of the bioactive peptides discovered in the protein sequences of *C. angulata* are di and tripeptides (Table S1). The dipeptide AE is the most frequently occurring DPP-IV inhibitory peptide in *C. angulata* proteins, especially in myosin heavy chain, paramyosin, and tropomyosin. A (alanine) belongs to the hydrophobic group of amino acids and is also occurring in considerable amount in oysters. E (glutamic acid), on the other hand, is observed to be abundant in oyster species [19,34]. However, it was observed that the activity of peptides containing the same amino acid residues but occurring in different position could exhibit different biological property. For example, the dipeptide AE which was characterized with DPP-IV inhibitory activity was also observed to demonstrate inhibitory effect against ACE when occurred in reversed form. This only means that the activity of a peptide could vary depending on the type of amino acid forming the peptide and its position in the protein sequence.

**Figure 3:**
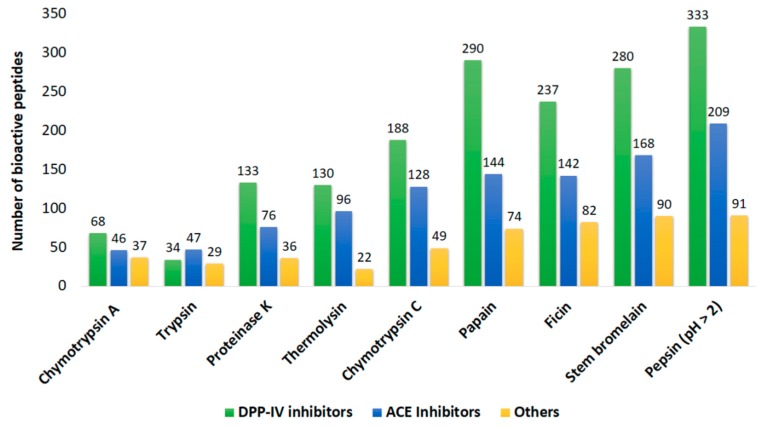
Total number of bioactive peptides released in silico by commercial enzymes through BIOPEP-UWM’s “Enzyme Action” tool (accessed on 6 March 2019). Other activities include antiamnestic, antibacterial, antioxidative, antithrombotic, neuropeptide, renin inhibitor, immunomodulating, stimulating, regulating, alpha glucosidase inhibitor, and activating ubiquitin-mediated proteolysis. DPP-IV: dipeptidyl peptidase IV.

Result of hydrolysis simulation using commonly used commercial enzymes is presented in Figure 3. Among the 9 enzymes used, pepsin (pH > 2) (EC 3.4.23.1) exhibited most of the DPP-IV and ACE inhibitory peptides theoretically, followed by stem bromelain (EC 3.4.22.32) and papain (EC 3.4.22.2). The effectiveness of these enzymes to release peptides with bioactivity depends mainly on its cleavage specificity. Pepsin has broad specificity and preferentially cleaves peptides with aromatic or carboxylic L-amino acid linkages, F and L at C-terminal location and to a lesser extent E linkages. However, it does not cleave at V, A, or G [35]. Stem bromelain exhibits strong cleavage preference for Z-R-R-I-NHMec among small molecule substrates while papain cleaves peptide bonds containing basic amino acids like arginine, lysine, and residues following phenylalanine [36,37]. The bioactive peptides released by different enzymes are listed in Table S2. With reference to in silico predictions, these three enzymes were chosen for use in the subsequent in vitro enzymatic hydrolysis.

### 2.3. In Vitro Hydrolysis of Oyster Proteins

Oyster protein isolate (OPI) was used as raw material for in vitro enzymatic hydrolysis. Among the three enzymes, pepsin gave the highest DH after 4 h of hydrolysis with a maximum value of 22.20 ± 0.97%, followed by papain and bromelain with 18.57 ± 0.61% and 17.86 ± 0.08%, respectively. The DH values of the three reactions increased rapidly from time 0 to 0.5 followed by a slower linear effect as hydrolysis time progresses (Figure S1). Generally, the rate of hydrolysis is faster during the initial stages of the reaction followed by a more static state and becomes steady when the highest DH is reached. Apparently, in this study, the reaction rate displayed an increasing trend even after 4 h which means that the highest DH for the three enzyme-catalyzed reactions was not yet achieved. One of the factors causing these slow reaction rates and low DH values is the low E/S ratio used in this study since a higher enzyme concentration would develop more cleavage activity. Moreover, the rate and extent of hydrolysis can also be affected by the secondary and tertiary structures of proteins. Some protein tertiary structures are sensitive to environmental conditions like acidic pH, making it unsusceptible to proteases and difficult to hydrolyze [38]. Hydrolysis condition, yield, degree of hydrolysis, and peptide content of the hydrolysates produced by different enzymes are summarized in Table 3.

PEH, which demonstrated the highest DH, also obtained the highest yield (84.69%) among the three hydrolysate samples. However, in terms of peptide content, the value obtained by PEH was observed to be very comparable to that of PAH, despite of the differences in their DH values. This might be due to the unequal volume of solution at 4 h of hydrolysis wherein the collection of sample aliquots was done. The high temperature used during papain hydrolysis could have also led to evaporation which causes the reduction of sample volume and increase in concentration of peptides in the sample solution. Nevertheless, results showed that the increase in DH can lead to the production of more small peptides and free amino acids.

**Figure 4:**
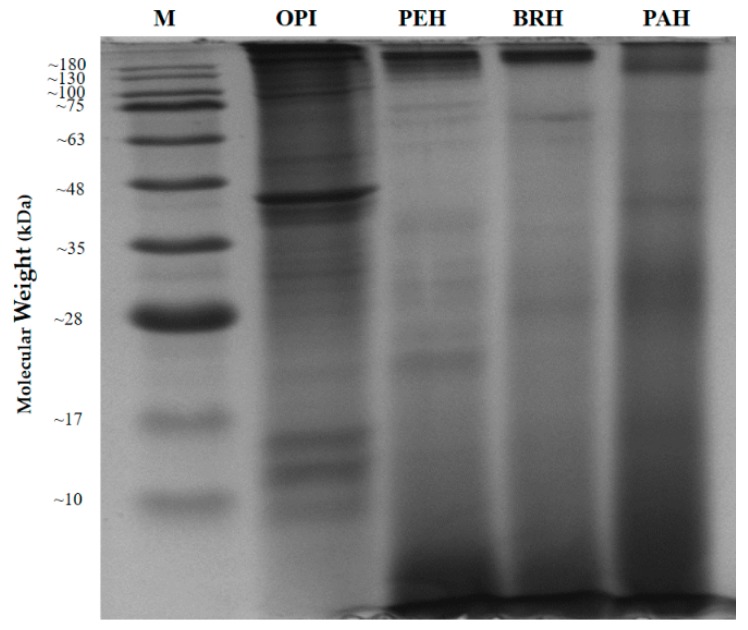
Peptide patterns of *C. angulata* protein hydrolysates by 15% SDS-PAGE. M: protein marker; OPI: oyster protein isolate; PEH: pepsin hydrolysate; BRH: bromelain hydrolysate; PAH: papain hydrolysate.

Based on the protein/ peptide patterns of *C. angulata* hydrolysates and fractions (Figure 4), all hydrolysates (PEH, BRH, and PAH) showed dispersion around and below 10 kDa, which were not observed in the OPI. Among the three hydrolysates, PEH has the highest concentration of low molecular weight peptides which is attributed to its high DH. The degradation of actin (previously identified by compiled proteomics techniques) to different extent are also evident in all hydrolyzed samples.

However, a band with the highest MW is observed to be visible even after hydrolysis, but appeared lighter in PAH than in PEH and BRH. This protein may be sensitive to high temperature applied during papain digestion. Moreover, the presence of light bands between 17 to 75 kDa indicates that there are still more protein substrates that were not cleaved even after 4 h of hydrolysis. This could be related to the low DH exhibited by the three enzymes. Overall, the electrophoretic pattern of *C. angulata* hydrolysate clearly supports the DH results, suggesting that pepsin’s ability to break down oyster proteins into smaller peptides is better than bromelain and papain.

### 2.4. Confirmation of Bioactivities Through In Vitro Tests

#### 2.4.1. ACE Inhibitory Activity

**Figure 5:**
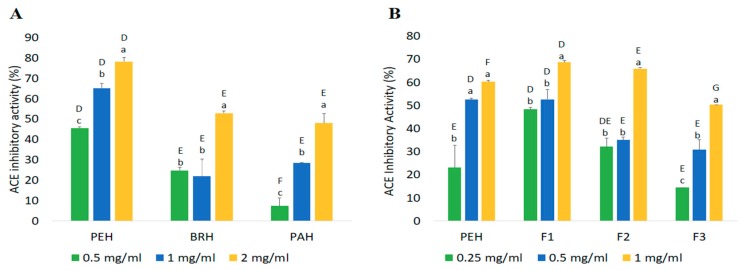
In vitro angiotensin I-converting enzyme (ACE) inhibitory activity of *C angulata* protein hydrolysates (**A**) and PEH fractions (**B**). Capital letters represent the significant difference (*p* < 0.05) among samples at specific concentrations; and small letters among concentrations within each sample. Each value (in percentage) represents the mean ± standard deviation (*n* = 3).

Angiotensin-I converting enzyme (ACE) is an enzyme responsible for the regulation of blood pressure. It converts angiotensin I into a potent vasoconstrictor angiotensin II and degrades the vasodilator, bradykinin, thus leading to an increase in blood pressure [39]. In this study, the potency of the three hydrolysates (PEH, BRH, and PAH) as inhibitors of ACE was evaluated. As shown in Figure 5A, all hydrolyzed samples exhibited an inhibitory activity against ACE which means that hydrolysis of proteins with pepsin, bromelain and papain were able to generate potent ACE inhibitory peptides. PEH displayed higher ACE inhibitory activity in all concentrations than BRH and PAH. The inhibition rates of the hydrolysate samples were observed to be dose-dependent except for BRH wherein a slight deviation was noticed at 1 mg/mL. Furthermore, the highest ACE inhibitory activity was noted in PEH prepared at 2 mg/mL with a value of 78.18 ± 2.19%, followed by BRH and PAH with 52.97 ± 1.01% and 42.65 ± 4.73%, respectively. It can be seen that PEH which have shown higher DH and peptide content than the other two hydrolysate samples also gave stronger inhibitory effect against ACE. One of the reasons for this is the high levels of free amino acids and smaller peptides liberated during hydrolysis that have ACE inhibitory properties. Basically, the biological activity of hydrolysates is influenced by the size, amount, composition of free amino acids and peptides, and the amino acid sequence [40]. This could also be associated to the cleavage specificity of pepsin, targeting the most bulky hydrophobic residues. The liberation of hydrophobic residues during pepsin hydrolysis results to their exposure to aqueous environment and susceptibility to reaction with different biomolecules, which leads to subsequent biological activities [41]. Overall, the results predicted in silico coincided with the results obtained in vitro with regards to the effectiveness of pepsin in releasing peptides with ACE inhibitory activity.

PEH was further separated into <1 kDa (F1), 1–5 kDa (F2), and >5 kDa (F3) fractions and their abilities to inhibit ACE activity were measured. The inhibition properties of PEH and peptide fractions against ACE followed a dose-dependent pattern in which an increase in concentration of peptides resulted in an increased inhibitory effect (Figure 5B). Result of ACE inhibitory activity assay revealed that F1 and F2 exhibited higher inhibitory activities (68.69 ± 0.82% and 65.95 ± 0.53%, respectively) compared to F3 (50.28 ± 0.09%) and PEH (60.32 ± 0.53%). Generally, peptides with very low MW are known to be most suitable for the formulation of therapeutic agents since these peptides can resist gastrointestinal digestion, thereby can be absorbed into the blood circulatory system in an intact form [42].

To test the inhibitory efficiency of crude PEH and fractions with respect to their MWs, the inhibitory efficiency ratio was calculated. Table 4 shows the peptide content, yield, and inhibition efficiency ratio of PEH, F1, F2, and F3. Result shows that F1 exhibited greatest efficiency in inhibiting ACE activity with an IER value of 217.05%/mg/mL compared to PEH and high MW fractions (F2 and F3), despite its low peptide content. This value is comparable to the IER of hard clam peptide fraction with a MW of 1360–1180 Da [43]. Analysis result indicates that products containing small peptides possess stronger ACE inhibitory activity. In addition, several studies have reported that those peptides with strong ACE inhibition are generally short peptides [44]. In most cases, peptides which contain 3–20 amino acids have greater potency as bioactive peptides than parent proteins [45].

The same with the unfractionated hydrolysate samples, the ACE inhibition properties of peptide fractions against ACE were observed to be dose-dependent in which an increase in the concentration of peptides resulted in increased inhibitory effect. Moreover, the inhibitory activity presented by PEH and its fractions is about half of the inhibitory activity of Captopril analyzed in this study (93.04%). Overall, results suggest that *C. angulata* proteins could be an important source of peptides that are capable of ACE inhibition.

#### 2.4.2. DPP-IV Inhibitory Activity

**Figure 6:**
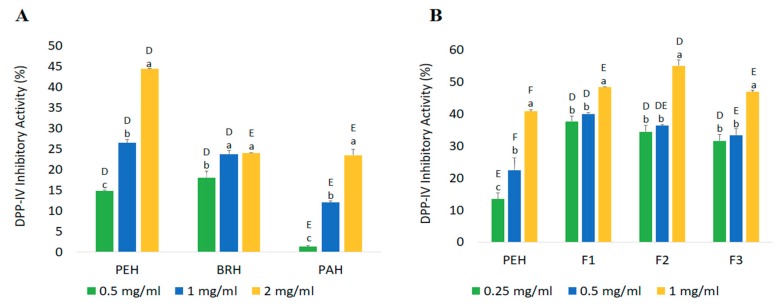
In vitro DPP-IV inhibitory activity of *C angulata* protein hydrolysates (**A**) and PEH fractions (**B**). Capital letters represent the significant difference (*p* < 0.05) among samples at specific concentrations; and small letters among concentrations within each sample. Each value (in percentage) represents the average of three samples ± standard deviation (*n* = 3).

Dipeptidyl peptidase-IV (DPP-IV) is a postproline-cleaving enzyme that causes the degradation of incretins GLP-1 and GIP, leading to an increase in the blood glucose level. In this study, the ability of PEH, BRH, and PAH to inhibit DPP-IV was measured in vitro. Figure 6A shows that all the hydrolysate samples produced by different enzymes were able to inhibit DPP-IV activity. The strongest inhibition was observed in PEH prepared at 2 mg/mL (44.37 ± 0.09%), followed by BRH (23.98 ± 0.07%) and PAH (23.44 ± 1.44%). These results are in agreement with the in silico predictions. This strong inhibitory activity of PEH can be related to the ability of pepsin to cleave peptides with aromatic amino acid linkages. Previous in silico studies have shown that DPP-IV inhibitory peptides usually have a branched-chain amino acid or an aromatic residue containing a polar group in the side chain (primarily W) at their *N*-terminal position and/or P residue located at their P_1_ [46]. In addition, the inhibitory activities of the hydrolysates were observed to be dose-dependent.

PEH, the hydrolysate with highest inhibitory activity, was subjected to fractionation and the DPP-IV inhibitory activities of the fractions were also examined. Results show that the ability of all fractions to inhibit DPP-IV activity was observed to increase with increasing concentration. Among the samples, F1 presented the strongest inhibitory activity at different concentrations. However, for 1 mg/mL, F2 (55.08 ± 1.98%) displayed higher inhibition value than F1 (48.42 ± 0.06%) (Figure 6B). Bioactivity of peptides does not only depend on molecular weight, but also on other factors like amino acid composition and sequences in their chemical structure [47]. Results revealed that low MW fractions demonstrated higher efficiency as inhibitors of DPP-IV than high MW fractions with reference to their IER values (Table 4). Moreover, the inhibitory activity of Diprotin A (98.83%) obtained in this study was about 50% higher compared to that of the PEH fractions. Nevertheless, the above findings suggest that peptides from *C. angulata* proteins can be a good alternative for bioactive peptides suitable for DPP-IV inhibition.

## 3. Materials and Methods

### 3.1. Materials

Portuguese oysters (*Crassostrea angulata*) were purchased from Penghu Island, Taiwan. They were packed in a box with ice and sent to the laboratory by freight transport. Pepsin (from porcine gastric mucosa), bromelain (from pineapple stem), and papain (from papaya) were obtained from Sigma-Aldrich (St. Louis, MO, USA). The angiotensin I converting enzyme (ACE) from rabbit lung (≥2 units/mg), *N*-(3-[2-furyl]-acryloyl)-phenylalanyl glycyl glycine (FAPGG), Dipeptidyl Peptidase IV (DPP-IV) from human recombinant (≥1 unit/mg), and Gly-Pro p-nitroanilide hydrochloride (≥99%) were also acquired from Sigma-Aldrich, USA. All chemical reagents used were of analytical grade.

### 3.2. Oyster Meat Preparation

Oysters (*C. angulata*) were manually shucked and the collected meat was washed with tap water and homogenized for 10 s using a food blender. The homogenized oyster meat was lyophilized for 48 to 72 h. It was then grounded into a fine powder (100 mesh). The resulting oyster powder were stored at −20 °C until further analysis.

### 3.3. Proteomics Techniques and In Silico Analysis

#### 3.3.1. Sodium Dodecyl Sulfate Polyacrylamide Gel Electrophoresis (SDS-Page) Analysis

*C. angulata* proteins were separated through SDS-PAGE as described by Laemmli [48]. Firstly, 1 mg of sample (dry weight, protein basis) was diluted in 1 mL sample buffer [0.5 M Tris–HCl (pH 6.8), glycerol, 10% (*w*/*v*) SDS, 0.5% (*w*/*v*) bromophenol blue, and β-mercaptoethanol]. The solution was then heated at 95 °C for 4 min and centrifuged at 4000× *g* for 15 min prior to loading. Electrophoresis was performed in a 12% running gel (ddH_2_O, 30% Acrylamide/Bis (37.5:1), 1.5 M Tris-HCl (pH 8.8), 10% (*w*/*v*) SDS, 10% (*w*/*v*) ammonium persulfate and TEMED) and 4% stacking gel (ddH_2_O, 30% Acrylamide/Bis (37.5:1), 0.5 M Tris-HCl (pH 6.8), 10% (*w*/*v*) SDS, 10% (*w*/*v*) ammonium persulfate and TEMED). Ten (10) µL of sample and 5 µL of standard (AccuRuler RGB prestained protein ladder, MaestronGen Inc., Taiwan) were loaded into each well of the gel. The voltage of power supply was set at 70 V for stacking gel and 110 V for running gel. After electrophoresis, the gel was stained with Coomassie Brilliant Blue for 30 min and subsequently destained with water/methanol/acetic acid (7/2/1, *v*/*v*/*v*) solution for 15 min with continuous shaking. The gel was then scanned using a gel image scanner and the MW of the visible bands was determined using the VisionCapt software (V16.08a, Vilber Lourmat, Paris, France).

#### 3.3.2. Gel Slice and In-Gel Digestion

Distinct protein bands from SDS-PAGE gel were sliced and subjected to in-gel digestion following the method of Shevchenko and group [49] with some modifications. The sliced bands were cut into small cubes measuring around 1 mm^3^. The gel sample was then placed in a siliconized eppendorf tube and spun down. Complete destaining was performed by subsequent addition of 50% and 25% acetonitrile/25 mM ammonium bicarbonate solution. Afterward, the gel sample was added with 100 µL DTE solution (50 mM dithioerythreitol/25 mM ammonium bicarbonate) and soaked for 1 h at 37 °C to break the disulfide bonds. After incubation, the mixture was spun down and the DTE solution was removed completely. The reduced gel sample was subjected to alkylation by incubating it with 100 µL of IAM solution (100 mM iodoacetamide/25 mM ammonium carbonate) for 1 h in the dark. After which, the gel sample was washed with 200 µL of 50% acetonitrile/25 mM ammonium bicarbonate for 15 min. The mixture was spun down and the buffer was pipetted out. Washing step was repeated 4 times to ensure that all buffers were removed completely. The gel sample was then soaked with 100 µL of 100% acetonitrile until it hardened and turned white. After removing all the acetonitrile, the remaining gel sample was dried for about 5 min in a SpeedVac concentrator. Digestion was performed by adding Lys-C/25 mM ammonium bicarbonate into the gel sample (1:50, enzyme:protein) followed by 3 h incubation at 37 °C. After that, the mixture was added with the same amount of trypsin and incubated at the same temperature for at least 16 h to complete the digestion. Prior to extraction, the enzyme was deactivated first by adding 50 µL of 50% acetonitrile/5% TFA to the mixture and sonicating it ten times (with 10 s interval). The mixture was centrifuged and the supernatant containing peptides was aspirated and transferred into a new tube. The remaining gel sample was extracted again following the above steps and the supernatant collected from the first and second extraction was combined and dried in a SpeedVac concentrator. The dried gel sample was then subjected to zip-tip purification prior to LC-ESI-MS/MS analysis.

#### 3.3.3. NanoLC–NanoESI–MS/MS Analysis

The MS/MS data of the peptide mixtures were acquired from the Institute of Biological Chemistry, Academia Sinica, Nangang District, Taipei, Taiwan. The liquid chromatographic separation was done using C_18_ column and separated using a segmented gradient in 60 min from 5% to 35% solvent B (acetonitrile with 0.1% formic acid) at a flow rate of 300 nl/min and a column temperature of 35 °C. Solvent A was prepared with 0.1% formic acid in water. The mass spectrometry analysis was performed in a data-dependent mode and full scan MS spectra was acquired in the orbitrap (*m*/*z* 350–1600) with the resolution set to 60K at *m*/*z* 400 and automatic gain control (AGC) target at 10^6^. The 20 most intense ions were sequentially isolated for CID MS/MS fragmentation and detection in the linear ion trap (AGC target at 10,000) with previously selected ions dynamically excluded for 60 s. Ions with singly and unrecognized charged state were also excluded.

#### 3.3.4. Mascot Database Search

The collected MS/MS raw data were converted into MGF files and subjected to Mascot database search to identify the proteins and/or peptides detected by MS. The data were searched against National Center for Biotechnological Information (NCBI) Database for metazoa (animals). Search parameters used were carbamidomethyl (C) and oxidation (M) for variable modifications, ±10 ppm for peptide mass tolerance, 2+, 3+, and 4+ for peptide charge, ±0.6 Da for fragment mass tolerance, 2 for maximum missed cleavages, ESI-trap for the instrument used, and trypsin for the enzyme applied. All peptide masses were obtained as monoisotopic masses.

The Mascot ion score was −10*Log (P), where P is the probability that the observed match is a random event. Protein scores were derived from ion scores as a non-probabilistic basis for ranking protein hits. Mascot search results presented the protein sequence coverage in percentage (%) indicating the sequence homology of identified tryptic peptides from oyster fractions to corresponding protein hits.

#### 3.3.5. BLAST Analysis of Oyster Proteins

Protein sequence of myosin essential light chain protein from *C. angulata* was compared to its *C. gigas* counterpart using BLAST (https://blast.ncbi.nlm.nih.gov/Blast.cgi), as described by Huang et al. [50], to determine the homology between the two proteins based on score, identities (%), positives (%), and gaps (%).3.3.6. BIOPEP-UWM analysis of bioactive peptides and enzyme cleavages.

The protein sequences obtained from the Mascot database search were analyzed using the BIOPEP database (http://www.uwm.edu.pl/biochemia/index.php/pl/biopep) to predict their bioactive peptide profile and enzyme cleavages. The activities, sequences, numbers and locations of bioactive peptides in protein sequences were determined using “profiles of potential biological activity” tool. The identified protein sequences were then examined through “enzyme action” tool wherein hydrolysis was simulated using different commercial enzymes available in the database. The theoretical peptides released by each enzyme were then directed to “search for active fragments” tool and the peptides with the highest number of potential bioactivities were selected for further analysis.

### 3.4. In Vitro Analyses

#### 3.4.1. Protein Isolation

*C. angulata* proteins were isolated using alkaline solubilization/ isoelectric precipitation method described by Huang et al. [50]. In brief, the lyophilized oyster meat was mixed with 0.1 M NaOH at a ratio of 1:20 (powder: NaOH, *w*/*v*) and stirred for 2 h at room temperature. The mixture was centrifuged for 10 min at a speed of 8000× *g* at 4 °C. The supernatant was collected and adjusted to pH 5.5 with 0.1 N HCl to precipitate the myofibrillar proteins. The pH modified mixture was centrifuged at 8000× *g* speed for 10 min at 4 °C and the collected precipitate was lyophilized and stored at −20 °C until further analysis. The yield of the protein isolate was calculated based on the dry weight of oyster protein isolate over the dry weight of oyster meat used in isolation multiplied by 100.

#### 3.4.2. Enzymatic Hydrolysis

Enzymatic hydrolysis was done following the combined methods of Dong et al. [19] and Jun et al. [51] with modifications. The oyster protein isolate (1 g, protein basis) was suspended in 100 mL deionized water. The homogenate was adjusted to 37 °C, pH 2 for pepsin; 50 °C, pH 7 for bromelain; and 65 °C, pH 7 for papain. The reaction started after adding the enzyme (1:100, *E*/*S*) and continued until 4 h. During hydrolysis, 1 mL aliquot of samples was taken at different time intervals (0, 0.5, 1, 1.5, 2, 2.5, 3, 3.5, and 4) for degree of hydrolysis determination. The enzyme activity was terminated by raising the temperature to 95 °C for 10 min. The sample was then cooled and centrifuged at 8000× *g* for 10 min. The recovered supernatant was neutralized to pH 7, lyophilized, and then stored at −20 °C for further analysis. The resulting dried hydrolysate was labeled as PEH (pepsin hydrolysate), BRH (bromelain hydrolysate), and PAH (papain hydrolysate).

#### 3.4.3. Degree of Hydrolysis

The degree of hydrolysis was calculated based on the amount of free amino groups using modified o-phthalaldehyde method by Charoenphun et al. [52]. The OPA reagent was prepared fresh by mixing 12.5 mL of 100 mM sodium tetraborate, 1.25 mL of 20% sodium dodecyl sulfate (SDS), 20 mg of OPA reagent dissolved in 0.5 mL methanol and 50 µL of β-mercaptoethanol. The final volume of the solution was adjusted to 25 mL by adding deionized water. In a 96-well microplate, 10 µL of hydrolysate sample/blank/standard was combined with 200 µL of OPA reagent and incubated at 37 °C for 100 s. The absorbance was read at 340 nm using a UV/Vis microplate spectrometer (SPECTROstar Nano, BMG LABTECH) and the amount of amino groups was quantified using Gly-Gly-Gly as standard. The total number of primary amino groups in the sample was determined by performing a complete acid hydrolysis using 6N HCl for 24 h at 110 °C. The % DH was calculated as follows:

DH (%) = [(NH_2_)_tx_ − (NH_2_)_t0_]/[(NH_2_)_total_ − (NH_2_)_t0_] × 100% (1)

where, (NH_2_)_tx_ is the amount of free amino groups at X min; and (NH_2_)_total_ is the amount of total amino groups by total acid hydrolysis. (NH_2_)_t0_ represents the amount of free amino groups at 0 min of hydrolysis.

#### 3.4.4. Fractionation

PEH was subjected to fractionation using Lefo Science-Spectrum Labs MAP-TFF Systems with a molecular weight cut-off of 5 and 1 kDa. The sample was dissolved in distilled water at 1% (*w*/*v*) concentration and placed in the ultrafiltration hollow fiber membrane. The recovered permeates, classified as F1 (<1 kDa), F2 (1–5 kDa), and F3 (>5 kDa) were then lyophilized and stored at −20 °C until further analysis. The yield of each fraction was calculated based on the dry weight of the fractions over the dry weight of the hydrolysate used multiplied by 100.

#### 3.4.5. Peptide Content Determination

The peptide contents of the hydrolysates and fractions were measured using the modified OPA method by Charoenphun et al. [52], previously used in measuring the degree of hydrolysis. The peptide content was quantified using Gly-Gly-Gly as standard.

#### 3.4.6. Angiotensin-I Converting Enzyme (ACE) Inhibitory Activity Assay

The ACE inhibitory activities of the protein hydrolysates and fractions were determined following the combined method of Raghavan and Kristinsson [53] and Udenigwe et al. [54]. In this method, *N*-[3-(2-furyl) acryloyl]-l-phenylalanyl glycyl glycine (FAPGG) was used as synthetic substrate for ACE. Each assay sample was dissolved in 50 mM Tris-HCl buffer (pH 7.5) containing 0.3 NaCl at a final assay concentration of 0.5, 1.0, and 2.0 mg protein/mL (for hydrolysates) and 0.25, 0.5, and 1 mg protein/mL (for fractions). In a 96-well microplate, 20 µL of sample was combined with 170 µL of 0.5 mM FAPGG solution and pre-incubated at 37 °C for 10 min. Buffer solution was used as control. Thereafter, 10 µL of 0.5 U/mL ACE (pre-heated at 37 °C for 10 min) was added to each well and the rate of decrease in absorbance at 345 nm was checked and recorded for 30 min at 1 min interval using a UV/Vis microplate spectrometer (SPECTROstar Nano, BMG LABTECH) preset to 37 °C. Captopril (1 mg/mL) was used as a reference inhibitor for the assay. The ACE inhibitory activity was calculated as:

ACE inhibitory activity (%) = [ΔAmin^−1^_(control)_ − ΔAmin^−1^_(sample)_/ΔAmin^−1^_(control)_] × 100% (2)

where ΔA min^−1^_(sample)_ is the ACE activity in the presence of peptides while ΔA min^−1^_(control)_ is the ACE activity in the absence of peptides.

#### 3.4.7. Dipeptidyl Peptidase IV (DPP-IV) Inhibitory Activity Assay

The inhibitory activities of the protein hydrolysates and fractions against the enzyme dipeptidyl peptidase-IV (DPP-IV) were determined following the combined methods of Lacroix and Li-Chan [55] and Zhang et al. [56]. Hydrolysate samples and fractions were dissolved in 100 mM Tris buffer (pH 8) to obtain a final assay concentration of 0.5, 1, and 2 mg protein/mL and 0.25, 0.5, and 1 mg protein/mL, respectively. In a 96-well microplate, 25 μL of assay sample was added with 25 μL of 1.6 mM Gly-Pro-p-nitroanilide and pre-incubated at 37 °C for 10 min. The mixture was then added with 50 µL of 0.008 U/mL DPP-IV (diluted with the same Tris-HCl buffer). Diprotin A (Ile-Pro-Ile) was used as reference inhibitor. Each sample was analyzed in triplicate and Tris-HCl buffer was used as blank. The positive control (DPP-IV activity in the absence of inhibitor) and negative control (no DPP-IV activity) was prepared using the same buffer solution in place of the sample and DPP-IV solution, respectively. The increase in absorbance per min was read in the UV/Vis microplate spectrometer (SPECTROstar Nano, BMG LABTECH) for 30 min at 37 °C and the rate of DPP-IV inhibition was calculated using the following equation:

DPP-IV inhibitory activity (%) = [1 − (A_s_ − A_b_)/(A_pc_ − A_nc_)] × 100 (3)

where A_s_, A_b_, A_pc_, and A_nc_ are the absorbance of the sample, blank, positive control, and negative control, respectively.

#### 3.4.8. Calculation of Inhibition Efficiency Ratio

The inhibition efficiency ratio of PEH and fractions was calculated based on the inhibitory activity (%) over the peptide content (mg/mL) of each sample.

### 3.5. Statistical Analysis

Data were presented as mean ± SD (standard deviation) for three replications for each sample. Data were analyzed using IBM SPSS (Statistical Package for Social Science,) version 20.0 (New York, NY, USA) for One-way Analysis of Variance (ANOVA) followed by Tukey’s post-hoc test to estimate the significance among the main effects at the 5% probability level.

## 4. Conclusions

The application of in silico technique provided a rapid and reliable information on the identification of bioactive peptides from *C. angulata* proteins, and in the determination of suitable enzyme for the generation of these peptides. The results have shown the correspondence between in silico prediction and in vitro confirmation. Based on the above findings, *C. angulata* protein hydrolysates can be a good source of peptides with ACE and DPP-IV inhibitory activities. Moreover, pepsin (pH > 2) demonstrated most promise in releasing bioactive peptides form *C. angulata* proteins both in silico and in vitro. Furthermore, fractionation enhanced the ability of the hydrolysate to inhibit ACE and DPP-IV activities. Overall, peptides from *C. angulata* proteins can be an alternative source of bioactive peptides capable of ACE and DPP-IV inhibition and can be used as a functional ingredient with pharmaceutical and nutraceutical applications. However, in vivo testing is highly suggested to ensure safety and stability of these peptides during gastrointestinal digestion.

## Figures and Tables

**Table 1:** Identified proteins from Portuguese oyster (*C. angulata*) meat and their characteristics.

Protein Name	Accession Number (NCBI)	Protein Score	Sequence Coverage (%)	Amino Acid Length	Molecular Weights from NCBI Database (kDa)	Molecular Weights Estimated from SDS-PAGE (kDa)
Myosin heavy chain, striated muscle isoform X1 *	XP_011442515.1	4670	48%	1901	222.66	130.00 (A)
						82.50 (B)
Paramyosin isoform X2 *	XP_011429256.1	2736	65%	851	102.21	82.50 (B)
Myosin heavy chain, striated muscle	EKC37566.1	789	20%	2001	229.67	55.14 (C)
						44.55 (D)
						40.75 (E)
						11.66 (F)
						9.15 (G)
						5.76 (H)
Actin *	EKC30049.1	1272	58%	351	41.80	44.55 (D)
Tropomyosin isoform X1 *	XP_019925727.1	579	70%	251	33.02	40.75 (E)
Hypothetical protein CGI_10010027	EKC40031.1	220	27%	151	20.58	11.66 (F)
Myosin regulatory light chain B, smooth adductor muscle isoform X2 *	XP_011417566.1	112	50%	151	18.63	9.15 (G)
						11.66 (F)
						5.76 (H)

* Selected proteins for in silico analysis (based on protein score and sequence coverage).

**Table 2:** Total number of potential bioactive peptides from oyster proteins predicted in silico using BIOPEP-UWM database (accessed on 6 March 2019).

Protein Name	Number of Potential Bioactive Peptides		
	ACE Inhibitor	DPP-IV Inhibitor	Other Activities *
myosin heavy chain, striated muscle isoform X1	721	1147	395
paramyosin isoform X2	294	517	147
actin	196	246	69
tropomyosin isoform X1	97	165	56
myosin regulatory light chain B, smooth adductor muscle isoform X2	83	104	39
**Total**	1391	2179	706

* Other activities include antiamnestic, antibacterial, antioxidative, antithrombotic, neuropeptide, renin inhibitor, immunomodulating, stimulating, regulating, alpha glucosidase inhibitor, activating ubiquitin-mediated proteolysis, etc.

**Table 3:** Hydrolysis conditions, yield, and peptide content of *C. angulata* protein hydrolysate. PEH: pepsin hydrolysate; BRH: bromelain hydrolysate; PAH: papain hydrolysate.

Hydrolysate	Hydrolysis Conditions				Maximum DH (%)	Yield * (%)	Peptide Content (mg/mL)
	E/S Ratio	Time (h)	pH	Temp. (°C)			
PEH	1:100	4	2	37	22.20 ± 0.97 ^a^	84.69	2.42 ± 0.06 ^a^
BRH	1:100	4	7	50	17.86 ± 0.08 ^b^	35.14	1.53 ± 0.03 ^b^
PAH	1:100	4	7	65	18.57 ± 0.61 ^b^	27.79	2.28 ± 0.03 ^a^

* The yield was calculated based on the dry weight of the lyophilized hydrolysate over the dry weight of the protein isolate used during hydrolysis. Different superscript letters have significantly different (*p* < 0.05) mean values.

**Table 4:** Inhibitory activity, peptide content, yield, and inhibitory efficiency ratio of pepsin hydrolysate and peptide fractions.

Fractions	Peptide Content (mg/mL)	Yield * (%)	Inhibition Efficiency Ratio ^a^ (%/mg/mL)	
			DPP-IV Inhibitory Peptides	ACE Inhibitory Peptides
PEH	2.42 ± 0.06 ^a^	84.69	16.86	24.92
F1	0.32 ± 0.06 ^c^	10.95	153.00	217.05
F2	0.45 ± 0.02 ^c^	2.05	123.26	147.60
F3	1.73 ± 0.00 ^b^	54.74	27.11	29.04

* Yield was calculated based on the dry weight of the lyophilized hydrolysate and fractions over the dry weight of the protein isolate and hydrolysate used during hydrolysis. ^a^ IER (inhibitory efficiency ratio) = % inhibition/peptide content. Different superscript letters represent significant difference between mean values (*n* = 3) at *p* < 0.05.

## Supplementary Materials

The following are available online at https://www.mdpi.com/1422-0067/20/20/5191/s1.
