# Supplementary material for: In Silico and In Vitro Assessment of Portuguese Oyster (Crassostrea angulata) Proteins as Precursor of Bioactive Peptides
Source: Int J Mol Sci. 2019 Oct 20;20(20):5191. doi: 10.3390/ijms20205191 (PMC6829514; doi:10.3390/ijms20205191)
Supplement: Supplementary file 1 [file ijms-20-05191-s001.pdf]

In Silico and *In Vitro* Assessment of Portuguese Oyster (*Crassostrea angulata*) Proteins as Precursor of Bioactive Peptides

Honey Lyn R. Gomez, Jose P. Peralta, Lhumen A. Tejano and Yu-Wei Chang

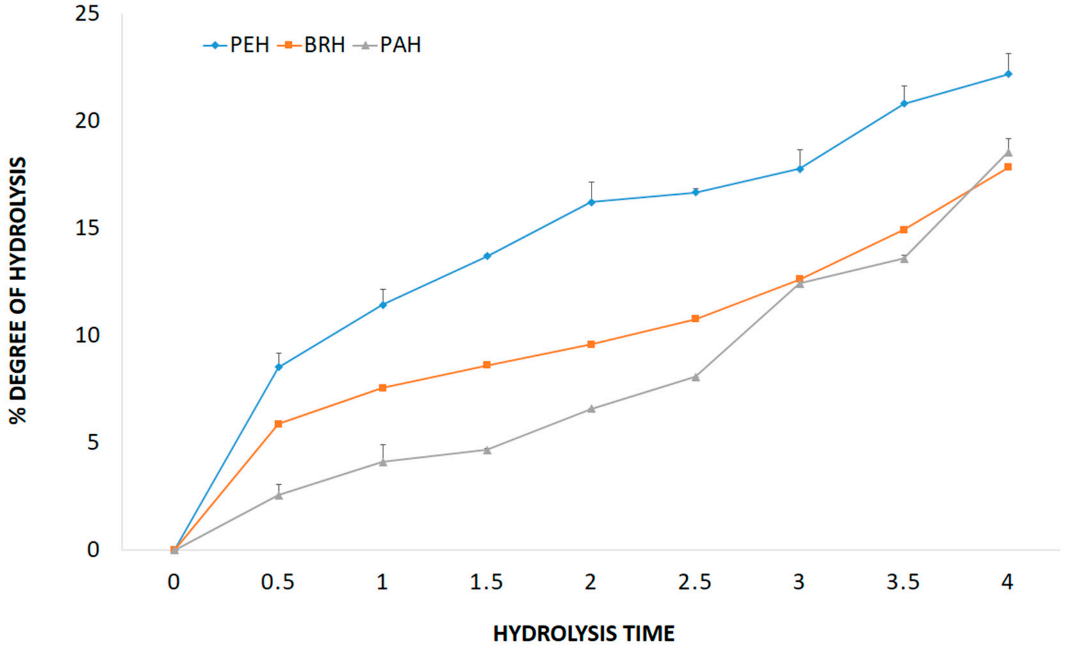

Figure S1. Degree of hydrolysis of *C. angulata* protein hydrolysates. PEH: pepsin hydrolysate; BRH: bromelain hydrolysate; PAH: papain hydrolysate.

Table S1. Summary of DPP-IV and ACE inhibitory peptide sequences discovered in oyster proteins using BIOPEP-UWM's "Profile of Potential Bioactivity" tool (accessed on March 06, 2019).

Protein	Bioactive Peptide Sequences	
	DPP-IV Inhibitory Peptides	ACE Inhibitory Peptides

Myosin heavy chain, striated muscle isoform X1	GP (1), PP (3), MP (1), VA (7), MA (4), KA (21), LA (16), FA (1), AP (3), PA (3), LP (1), VP (3), LL (8), VV (3), HA (3), IPA (1), IP (3), SP (2), FP (2), RP (1), KP (5), HP (1), GA (8), IA (9), RA (14), EP (1), NP (3), TA (6), FL (3), WV (1), HL (4), EK (26), AL (14), SL (12), GL (3), VR (6), WRS (1), AA(18), ILAP (1), WR (1), WK (1), WL (3), WQ (2), WW (1), MW (1), WE (1), YT (2), AD (16), AE (32), AF (5), AG (12), AH (3), AS (9), AT (3), AV (9), AY (4), DN (6), DP (5), DQ (5), DR (8), EG (7), EH (3), EI (12), ES (12), EV (8), EW (1), EY (3), FN (2), FQ (4), FR (2), GE (4), GF (6), GG (7), GH (2), GI (9), GV (3), GW (1), GY (2), HE (5), HF (2), HH (1), HI (1), HR (1), HS (2), HY (1), IH (2), II (3), IL (4), IM (1KR (12),), IN (10), IQ (5), IR (7), KE (15), KF (3), KG (8), KI (6), KK (31), KS (14), KT (10), KV (15), KW (1), KY (3), LH (3), LI (4), LM (4), LN (13), LT (6), LV (8), ME (7), MF (3), MG (4), MH (2), MI (4), MK (7), ML (5), MN (2), MQ (6), MR (5), MV (1), MY (2), NA (11), ND (3), NE (13), NF (1), NG (1), NH (3), NL (8), NM (1), NN (4), NQ (6), NR (5), NT (3), NV (4), NW (2), NY (3), PF (1), PG (2), PH (2), PI (2), PK (3), PM (2), PN (5), PQ (1), PS (1), PT (1), PV (2), PY (1), QA (13), QD (7), QE (11), QF (4), QG (5), QH (1), QI (9), QL (15), QN (3), QQ (8), QS (8), QT (7), QV (6), QW (2), QY (2), RG (3), RH (1), RI (11), RK (12), RL (15), RM (4), RN (7), RR (10), SF (4), SH (1), SI (8), SK (9), SV (6), TD (4), TE (7), TF (2), TG (2), TH (1), TI (3), TK (6), TL (7), TN (1), TQ (2), TR (6), TS (6), TT (3), TV (7), TY (5), VD (6), VE (12), VF (1), VG (3), VH (2), VI (3), VK (10), VL (11), VM (1), VN (5), VQ (8), VS (5), VT (3), VY (1), YA (4), YD (2), YE (2), YF (1), YG (2), YH (1), YI (2), YK (6), YL (4), YN (1), YQ (4), YR (3), YS (6), YY (1)	RL (15), IR (7), LKL (1), AVP (1), FGK (2), AKK (1), RY (3), LY (6), IY (4), VF (1), MF (3), KW (1), MY (2), LVL (2), RF (1), VY (1), HY (1), FP (2), GKP (1), IPA (1), VAA (2), GY (2), PR (2), VSP (1), LAA (3), IRA (1), YL (4), LF (2), YG (2), FY (1), AY (4), AIP (1), LQQ (1), GP (1), LKP (1), VK (10), IA (9), LKA (1), LAP (1), MNPPK (1), GW (1), IP (3), RP (1), AF (5), AP (3), LA (16), KR (12), VP (3), RA (14), YA (4), AA (18), GF (6), FR (2), IF (2), VG (3), IG (3), GI (9), GM (3), GA (8), GL (3), AG (12), GH (2), HL (4), GR (2), KG (8), FG (5), DA (5), GS (2), GV (3), MG (4),GQ (5), GK (14), GT (2), GE (4), GG (7), QG (5), SG (5), LG (6), GD (4), TG (2), EG (7), EA (30), NG (1), PG (2), IAK (2), VR (6), PAP (1), VAV (1), MNP (1), NPP (1), PPK (1), MKY (1), NKL (2), LIY (1), QK (7), DG (4), NY (3), NF (1), SF (4), KY (3), KF (3), KL (18), YK (6), NK (11), RR (10), AR (5), KA (21), VIY (1), EY (3), KP (5), MDLA (1), RIY (1), FAL (1), IAE (1), EI (12), IE (16), EV (8), VE (12), TE (7), LQ (15), LN (13), PT (1), TQ (2), AH (3), PP (3), PQ (1), EW (1), ME (7), EK (26), KE (15), HP (1), PH (2), HK (3), VAF (1), IQY (1), TF (2), AI (3), AFLL (1), RYQ (1), VNP (1), VKP (1), AV (9), ASL (1), LEE (9), GKV (1), AFL (1), IVQ (1), AQL (1), MPP (1), LGI (1), LEK (6), DF (3), DM (3), FQ (4), YE (2), IL (4), YH (1), AEL (1), MW (1), WL (3), RG (3), ST (6), YN (1), AGS (1), DFG (1), LR (10), RRR (1), RVR (1), IQW (1)
paramyosin isoform X2	VA (4), KA (6), LA (11), FA (2), PA (1), LL (2), VV (2), HA (1), SP (1), GA (2), IA (2), RA (11), TA (3), FL (1), EK (6), AL (13), SL (7), GL (2), VR (5), AA (16), AD (4), AE (26), AF (2), AG (2), AS (7), AT (1), AV (1), DN (4), DQ (4), DR (3), EG (4), EH (1), EI (5), ES (9), ET (8), EV (7), EY (2), FQ (2), GE (3), GI (1), HR (2), II (2), IN (4), IQ (3), IR (5), KE (5), KG (1), KH (1), KI (3), KK (2), KR (9), KS (5), KT (4), KV (3), KY (3), LH (1), LI (1), LN (5), LT (10), LV (5), ME (2), MM (1), MN (1), MQ (1), MR (3), MY (1), NA (14), ND (3), NE (2), NF (1), NG (1), NL (10), NM (1), NN (2), NQ (2), NR (7), NT (1), NV (1), NY (1), PS (1), QA (5), QD (2), QE (7), QH (2), QI (5), QL (17), QN (3), QS (2), QT (3), QV (6), QY (1), RG (2), RH (1), RI (5), RK (10), RL (15), RM (1), RN (3), RR (5), SI (2), SK (2), SV (4), SY (1), TD (1), TE (8), TF (1), TI (2), TK (6), TL (2), TN (2), TQ (4), TR (9), TS (2), TV (3), TY (2), VD (3), VE (3), VI (2), VK (2), VL (1), VN (3), VQ (5), VS (3), VT (4), VY (2), YD (3), YE (4), YK (4), YL (1), YM (1), YN (1), YR (3)	RL (15), IR (5), RY (3), MY (1), RF (2), VY (2), PR (1), LAA (4), YL (1), LF (1), VK (2), IA (2), LKA (1), AF (2), LA (11), KR (9), RA (11), AA (16), IG (1), IG (1), GM (1), GA (2), GL (2), AG (2), GR (1), KG (1), DA (10), GS (1), GQ (1), GK (1), GT (1), GE (3), SG (1), LG (1), GD (1), EG (4), EA (12), NG (1), IAK (1), VR (5), LTF (1), QK (2), NY (1), NF (1), SY (1), KY (3), KL (5), YK (4), NK (2), RR (5), AR (7), KA (6), LVE (1), EY (2), EI (5), IE (3), EV (7), VE (3), TE (8), LQ (11), LN (5), TQ (4), ME (2), EK (6), KE (5), TF (1), AI (2), AV (1), LEE (3), VQV (2), AQL (2), VTR (1), DY (2), FQ (2), YE (4), MM (1), AEL (6), RG (2), ST (3), YN (1), LR (13)

actin	GP (1), PP (1), VA (4), MA (1), KA (2), LA (3), FA (1), AP (5), PA (1), LP (2), VP (1), LL (1), HA (1), SP (1), FP (1), RP (1), HP (1), YP (1), GA (1), IA (2), RA (2), NP (1), TA (2), QP (1), FL (1), EK (5), AL (4), SL (4), AA (2), PL (1), WI (2), MW (1), AD (1), AE (1), AG (3), AS (5), AT (1), AV (2), DN (1), DR (1), EG (1), EH (2), EI (2), ES (2), ET (2), EY (1), FN (1), FQ (2), FR (1), GF (1), GG (2), GI (6), GV (2), GY (2), HE (1), HH (1), HR (1), HT (2), IH (1), IL (3), IM (3), IN (1), IQ (1), IR (1), IW (1), KE (2), KI (3), KR (1), KS (1), KV (1), KY (2), LM (1), LN (1), LT (4), LV (1), ME (2), MF (1), MG (2), MK (3), MQ (1), MR (1), MV (1), MY (2), NE (2), NG (1), NR (1), NT (1), NW (1), PG (1), PH (1), PI (2), PK (1), PQ (1), PS (3), PV (1), QA (1), QE (2), QG (1), QI (1), QQ (1), QS (1), QT (1), QV (1), RG (2), RH (1), RK (3), RL (1), RM (1), SF (2), SH (1), SI (4), SK (2), SV (1), SY (2), TD (1), TE (2), TF (3), TG (1), TI (2), TL (1), TM (2), TN (1), TQ (1), TR (1), TS (1), TT (5), TV (2), TY (1), VD (1), VF (1), VG (3), VH (1), VI (3), VL (3), VM (1), VS (1), VT (1), VW (1), WD (1), WH (1), YA (3), YD (1), YE (2), YL (1), YN (1), YS (2), YV (3)	RL (1), IR (1), FQP (1), ALPHA (1), IVGRPRHQG (1), IWHHT (1), GYALPHA (1), YALPHA (1), GRP (1), LY (2), IY (1), VF (1), MF (1), MY (2), VW (1), RF (1), FP (1), VAA (1), VAP (1), GY (2), PR (1), LNP (1), YL (1), LF (1), FY (1), YP (1), GP (1), PL (1), IW (1), IWH (1), VFPS (1), IA (2), LAP (1), RP (1), AP (5), LA (3), KR (1), VP (1), RA (2), YA (3), AA (2), GF (1), FR (1), VG (3), IG (3), GI (6), GM (3), GA (1), AG (3), GR (3), DA (1), GS (2), GV (2), MG (2), GQ (2), GT (1), HG (1), GG (2), QG (1), SG (6), LG (1), GD (4), TG (1), EG (1), EA (4), NG (1), PG (1), QK (2), DG (2), SY (2), SF (2), KY (2), KL (1), KA (2), CF (1), EY (1), VWIG (1), IAP (1), EI (2), IE (1), TE (2), LN (1), TQ (1), PP (1), PQ (1), ME (2), EK (5), KE (2), HP (1), PH (1), TF (3), AI (2), MYPGIA (1), GIHETTY (1), EKSYELP (1), AV (2), ASL (1), IVGRPR (1), FQPSF (1), IYEGY (1), HQG (1), HHT (1), ALP (1), YVA (2), FYN (1), EAP (1), LEK (1), DY (1), DF (1), DM (1), FQ (2), YV (3), YE (2), IL (3), MW (1), SGP (1), RG (2), FQPSF (1), ST (2), YN (1), LR (1), FQPSF (1), IYEGY (1)
tropomyosin isoform X1	VA (1), KA (3), LA (3), FA (1), LL (2), VV (1), GA (1), IA (2), RA (4), NP (1), TA (2), EK (6), SL (2), AA (3), AD(2), AE (10), AG (1), AS (6), AT (2), DQ (2), DR (2), EI (1), ES (1), ET (1), EV (2), GI (1), GY (1), HS (1), IN (1), IQ (2), IR (1), KE (3), KH (1), KI (1), KK (4), KT (2), KV (2), KY (2), LI (1), LN (2), LT (3), ME (3), MI (1), MK (1), MY (1), NA (2), ND (1), NE (2), NL (2), NN (2), NR (3), QA (1), QD (1), QE (2), QL (5), QQ (1), QS (1), QT (3), QV (1), RI (1), RK (2), RL (4), RM (1), RN (1), RR (1), SI (2), SK (2), SV (1), SY (1), TD (1), TE (5), TF (1), TI (1), TK (1), TL (1), TQ (1), TR (2), TS (1), TV (2), VD (2), VL (2), VN (1), VQ (1), VS (1), VY (1), YD (1), YE (2), YK (1), YQ (1)	RL (4), IR (1), RY (1), MY (1), VY (1), GY (1), IA (2), LA (3), RA (4), AA (3), IG (1), GI (1), GA (1), AG (1), DA (1), EA (11), QK (3), SY (1), KY (2), KL (4), YK (1), RR (1), AR (2), KA (3), IAE (1), EI (1), IE (1), EV (2), TE (5), LQ (4), LN (2), TQ (1), ME (3), EK (6), KE (3), TF (1), AI (2), LEE (3), LEK (1), DM (1), YE (2), ST (1), LR (1)
myosin regulatory light chain B, smooth adductor muscle isoform X2	GP (1), KA (1), LA (1), FA (1), AP (2), LL (1), HA (1), APG (1), IP (1), TP (1), GA (1), RA (2), FL (1), EK (1), SL (3), VR (1), AA (1), PL (1), AD (1), AE (1), AF (2), AS (2), AT (2), DN (1), DP (1), DQ (1), EG (2), EY (1), FN (1), GF (1), GG (2), GH (1), GI (2), HT (1), II (1), IN (2), IQ (2), KE (3), KF (2), KG (2), KI (1), KT (1), KV (1), LI (1), LN (1), LT (1), MF (3), MG (1), MI (1), MK (1), ML (1), MR (1), MY (1), NA (1), ND (1), NF (2), NM (1), NQ (1), NR (1), NV (1), PG (1), PI (1), QE (1), QN (1), QQ (2), QT (1), RM (1), RN (1), SI (1), TD (1), TE (1), TF (1), TH (1), TK (2), TL (1), TM (2), TR (1), TS (1), VG (1), VI (1), VL (1), YE (1), YI (1)	RY (1), MF (3), MY (1), PR (1), LF (1), FNQ (1), GPL (1), GP (1), PL (1), IP (1), AF (2), AP (2), LA (1), RA (2), AA (1), GF (1), VG (1), IG (2), GI (2), GA (1), GH (1), KG (2), DA (1), GS (2), MG (1), GK (3), GT (1), GG (2), LG (2), GD (1), EG (2), EA (5), PG (1), VR (1), QK (1), DG (2), NF (2), KF (2), KL (2), KA (1), EY (1), IE (1), TE (1), LN (1), EK (1), KE (3), TF (1), AI (1), LNF (1), FDK (1), EAP (1), DY (1), TP (1), YE (1), ST (2), LR (1), LDY (1)

Table S2. Summary of bioactive peptides theoretically released by pepsin, stem bromelain, and papain.

Protein Name	Pepsin (pH > 2)	Stem Bromelain	Papain
myosin heavy chain, striated muscle isoform X1	PG (3), PP(2), VA (7), PA (2), HA (1), RL (18), HL (9), RY (2), IY (8), VF (2), HY (2), IPA (2), IRA (1), RA (33), VK (20), IA (16), IF (2), VG (6), IG (2), SG (5), PG (2), VR (2), PPK (2), SF (9), IE (16), VE (24), PT (2), HK (3), VL (22), IL (12), SE (10), SL (10), WL (3), WQ (2), WE (1), HE (2), IM (1), IN (8), IQ (5), PK (2), PM (2), PN (2), PY (1), RG (1), RK (9), RM (2), RN (4), SK (6), VD (6), VM (1), VN (5), VQ (8), VS (1), VT (3), WL (3), RG (1), ST (3)	PG (4), YG (3), MA (1), KA (26), HA (2), IR (5), HL (3), MF (1), IPA (2), PR (1), YL (9), IA (1), KR (16), YA (9), IG (1), KG (12), DA (1), MG (2), QG (2), EG (8), EA (13), NKL (2), DG (4), KF (2), KL (7), IE (1), EV (8), PT (2), EL (7), KF (2), EF (8), IV (1), IL (9), YQL (1), IA (1), WL (2), DR (2), ES (5), ET (5), HR (1), HS (1), KS (9), KT (4), KV (8), MF (1), ML (3), MR (3), MV (1), NA (5), NL (2), NR (4), NT (1), NV (1), PF (1), PS (1), QA (10), QF (2), QG (2), QL (9), QS (2), QT (1), QV (4), YS (5), DF (1), KCL (1)	PG (5), IR (5), HHL (3), EEE (1), AVP (1), AKK (1), MF (2), HY (2), YL (9), AY (6), IP (2), AF (10), KR (2), VG (2), AG (24), KG (8), MG (2), QG (10), EG (6), VR (10), NKL (2), DG (1), SF (3), KF (4), KL (2), NK (1), AR (5), EI (2), IE (1), EV (2), PT (2), ME (2), EL (4), KD (1), EF (4), VL (12), IL (3), II (2), EE (5), SE (1), AL (14), SL (3), WL (2), AD (1), AE (9), AS (3), AT (2), AV (2), DP (1), EH (1), ES (1), ET (1), HE (1), IN (3), KI (1), KS (1), KT (2), KV (1), ML (1), MR (1), MV (1), NE (2), NH (1), NL (1), NT (1), NV (1), NW (1), PK (1), QD (1), QF (8), QI (1), QL (15), QS (1), QT (7), QV (1), SK (1), SV (1), VT (1), ASL (1), DF (1), AEL (1), KCL (1)

paramyosin isoform X2	VA (4), HA (1), RL (22), RY (3), RF (2), VY (6), RA (24), VK (4), IA (4), SG (1), VR (4), SY (2), IE (3), VE (6), RHQ (1), VL (2), SE (2), SL (4), HR (1), IN (3), IQ (3), RG (4), RK (7), RN (3), SK (2), VD (3), VN (3), VQ (5), VT (4), ST (1)	KA (8), IR (15), KR (10), KG (2), DA (5), EG (8), EA (6), NG (2), KL (5), EV (10), EL (13), EF (2), DR (2), ES (4), ET (6), KS (4), KT (2), KV (3), MR (1), NA (7), NL (5), NR (10), PS (1), QA (4), QL (11), QS (1), QT (1), QV (5), YR (2)	IR (10), AF (4), KR (10), AG (4), EG (8), NG (2), VR (2), QK (1), KL (2), AR (6), EV (1), EK (4), EL (5), VKL (1), KD (1), VL (2), EE (2), AL (13), SL (1), AD (2), AE (2), AS (1), AT (1), DN (1), ES (1), ET (3), EV (1), KT (1), KV (1), NL (2), NR (2), QD (1), QE (1), QH (1), QI (1), QL (17), QT (3), QV (4), VD (1), VN (1), AEL (1)
actin	PG (5), VA (4), VP (2), RL (2), IWHHT (1), IY (2), VF (2), VW (4), RF (1), PL (2), IA (4), VG (6), IG (2), HG (1), SG (5), SY (4), SF (2), CF (1), IE (1), PQ (2), HP (2), PHA (1), VL (3), IL (9), SL (3), IM (3), IQ (1), PK (1), PS (1), RG (4), RK (1), RM (1), SK (1), VD (1), VL (3), VM (1), VT (1), WD (1), SF (1)	PA (1), PR (2), PL (2), IA (4), KR (2), YA (9), IG (1), MG (2), EA (1), DG (1), PHA (1), IV (4), IL (6), DR (1), ET (1), HR (1), HT (1), MV (1), NR (2), NT (1), PS (2), QS (1), QV (1), YS (1), YV (2), HQG (1), DF (1)	AP (2), PR (1), IG (1), AG (6), MG (2), QG (2), AI (1), SG (2), DG (1), PH (2), VL (2), IL (3), AL (4), SL (2), AT (1), ET (1), QT (1), ASL (1), DF (1)
tropomyosin isoform X1	VA (1), RL (8), RY (1), VY (3), RA (9), IA (4), SY (2), RR (2), IE (1), VL (4), SE (3), SL (2), IQ (2), RK (2), RN (1), SK (1), VD (2), VN (1), VQ (1)	KA (2), IR (5), IA (2), EA (6), KL (3), EV (2), DR (1), KV (2), NL (1), NR (4), QT (1), QV (1)	IR (2), AG (2), QK (1), KL (1), YK (2), AR (2), EK (2), KD (1), IR (3), EE (3), SL (1), AE (4), AS (1), AT (2), NN (1), NR (4), QL (5), QT (3), QV (1)
myosin regulatory light chain B, smooth adductor muscle isoform X2	PG (5), PL (2), VG (2), IE (1), VL (2), SL (2), IQ (1), RN (1), ST (1)	PG (5), MF (4), PL (2), KG (2), EA (1), DG (1), NF (2), KF (4), KL (1), NA (1), NV (1), QT (1)	MF (2), PL (2), AF (4), VG (2), KG (2), DG (1), NF (2), KF (4), KL (1), APG (1), SL (1), AE (1), AT (1), QT (1), VI (1), ST (1)
